# Neurobiological correlates of the social and emotional impact of peer victimization: A review

**DOI:** 10.3389/fpsyt.2022.866926

**Published:** 2022-08-01

**Authors:** Ana Cubillo

**Affiliations:** ^1^Jacobs Center for Productive Youth Development, Zurich Center for Neuroeconomics, University of Zürich, Zurich, Switzerland; ^2^Department of Child and Adolescent Psychiatry, University Psychiatric Clinic Basel, Basel, Switzerland

**Keywords:** peer victimization, social processing, adolescents, neurobiology, reward

## Abstract

Peer victimization is very common during late childhood and adolescence. Despite the relatively reduced number of studies, the neurobiological underpinnings of the negative impact of peer victimization experiences have received increasing attention in recent years. The present selective review summarizes the most recent available evidence and provides a general overview of the impact of peer victimization experiences on social processing and decision-making at the neurobiological level, highlighting the most pressing areas requiring further research. Three key cognitive areas show a clear negative impact of peer victimization and bullying experiences: social valuation processing, reward and reinforcement learning and self-regulation processes. Victims show enhanced activation in key regions of the limbic system including the amygdala, rostral and dorsal anterior cingulate cortices, suggestive of enhanced sensitivity to social stimuli. They also show enhanced recruitment of lateral prefrontal regions crucially involved in cognitive and emotional regulation processes, and abnormal reward-related striatal function. The presence of psychopathology is a complex factor, increased as a consequence of peer victimization, but that also constitutes vulnerability to such experiences.

## Introduction

**Figure 1:**
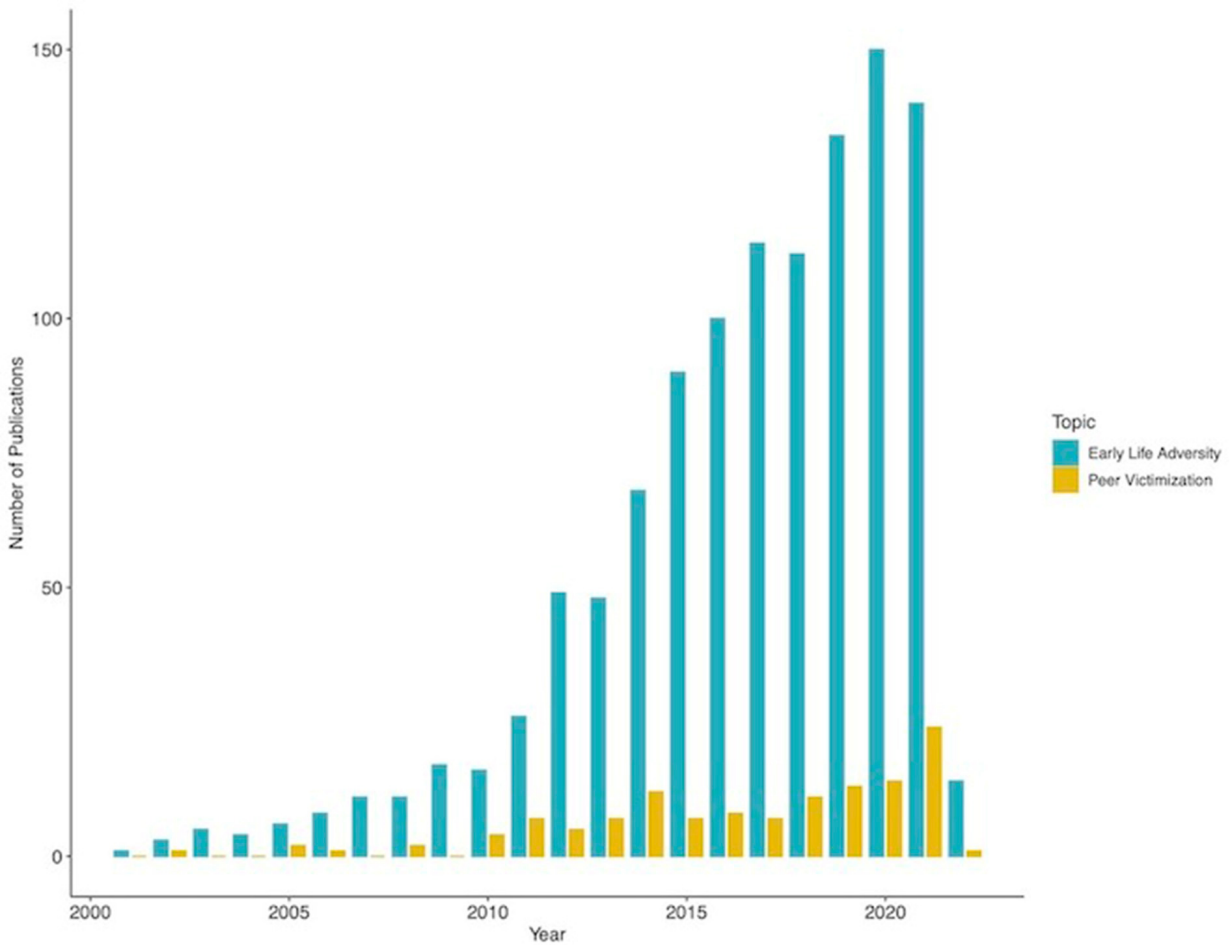
Comparison of the number of publications on neural mechanisms of peer victimization. The figure shows the number of publications displayed in a search in pubmed with the words “Peer victimization” and “brain” relative to the number of publications on “Early Life Adversity” and “Brain”. Search data: 25 January 2022.

For better and worse, social interactions play a large role in human development and well-being. Humans are social organisms by nature, requiring social contact for survival and reproduction. Human offspring rely on parental or other adult's care for survival for an extended period of time because their physical and cognitive development extends over decades. This allows the human species to develop complex behavior and thinking patterns, but also makes them highly dependent on the positive or negative influences of relevant others during key sensitive and vulnerable periods. Social interactions during early childhood are focused primarily on parents or caregivers, slowly shifting through late childhood and adolescence toward peers. This shift is accompanied by progressive maturation in neural systems supporting social processing (1, 2). Any adverse social experiences during these early phases of life can have cascade effects and substantially influence subsequent development. While the role of early adverse events such as parental abuse or neglect on neural development has been widely investigated for decades, the effects of acute and chronic peer victimization or bullying have more recently received much-needed attention as well (1) (Figure 1). Given the high prevalence and the pervasive, long-lasting impact, it is still surprising the relatively reduced number of publications focused on the neurobiological mechanisms of early experiences of peer victimization. However, the neural mechanisms of peer victimization experiences have received increasing attention in the last few years. It is therefore necessary to review what we now know and highlight the areas where more research is urgently needed. This article reviews the most recent evidence on the impact of peer victimization on brain function during social learning and decision-making and highlight the most pressing aspects for future research studies.

Peer victimization can take the form of relational victimization (social exclusion, rumor spreading) and/or physical victimization (bullying, punching). These two forms of victimization have been shown to be highly correlated, with polyvictimization, conceptualized as the simultaneous exposure to different types of abuse, being highly common (3). Peer victimization and bullying are frequent in late childhood and adolescence, with prevalence estimates between 35 and 49% (4, 5). Such high frequency does not imply it should be treated as a “harmless rite of passage”. On the contrary, similar to the negative and long-lasting impact of early experiences of neglect or abuse on life outcomes (6–10), there is now compelling evidence for pervasive adverse short- and long-term effects of peer victimization on physical and somatic symptoms, psychological health (increase rates of anxiety, depression and suicidality), inflammation markers, stress response, social relationships, academic and occupational achievements or cognitive function (11–19).

Recent studies have started to shed light on the neurobiological correlates of peer victimization experiences, showing that experiences of social rejection, exclusion or bullying may trigger enhanced activation on or connectivity in regions supporting valuation and salience processes. The most recent evidence suggests peer victimization enhances individual's sensitivity to social stimuli (20–25), which together with altered reward and reinforcement learning processing (20, 26–28) and the difficulties in emotion and behavioral regulation lead to the increased need to engage regulatory circuits in order to implement behavioral, cognitive and emotional adaptations (29–31), not always successfully. Given the key role of peer interactions in socio-emotional development during childhood and adolescence, this selective review focuses on the associations between early experiences of peer victimization and bullying and altered neurobiological function during social valuation, and social decision-making, summarizing the most recent findings. A summary of the main results of the above studies, which are reviewed here, can be seen in Table 1.

**Table 1:** Summary of main findings of studies on peer victimization combining brain imaging techniques and behavioral paradigms on social, emotional and cognitive control processes.

**Reference**	**Population**	**Ages**	**Design**	**Methods**	**Key results**
Asscheman et al. (32)	Children (*N =* 55, 0F); low preferred by peers *N =* 27, high preferred by peers *N =* 28	8-12	Longitudinal peer preference assessment, Cross-sectional imaging WB + ROI	Cyberball	**a) Behavior:** low preferred boys less satisfaction after inclusion **b) Imaging** Exclusion: Low preferred > high preferred dlPFC, SMG • No differences in ROI dACC analysis
Cara et al. (29)	Children typically developing (*N =* 37, 12F)	9–14	Cross-sectional WB	Change task (modified Go/NoGo)	**a) Behavior** • No association exposure to violence and performance **B) Imaging** • Exposure to lifetime violence associated with reduced activation in dACC, L IFC, R SFG, bilateral precentral cortex, L insula • Exposure to last year violence associated with reduced activation in dACC, precentral gryus, bilat SFG, bilat MFG, L SPL • Negative association between dACC, Insula, SPL activation and progressive task performance deterioration
Cisler et al. (20)	Assault victims (*N =* 30, all F), and typically developing (*N =* 30, all F)	11–17	Cross-sectional ROI	Social and non-social Reinforcement Learning (three-arm bandit) tasks Emotion processing task	**a) Behavior** • No differences in social vs. non-social; no differences between groups **b) Imaging** • Salience network (dACC, Insula) identified at ICA analysis weaker encoding of negative PE in victims vs. TD in both tasks. ***This association varied as a function of trauma***. • Increased activation dACC, Insula during fear faces in high victimized group
Ethridge et al. (26)	Young adults exposed to victimization (*N =* 61, 54F)	18–25	Cross-sectional	Doors task EEG study	• Past-year relational but not physical victimization was associated with smaller neural response to gain, indicative of blunted reward response
Fowler et al. (33)	Study 1: healthy adolescents (*N =* 33, 20F) Study 2: Adolescents (*N =* 26, all F) with (*N =* 17) and without (*N =* 9) past exposure to peer victimization	11–16 14–16	Cross-sectional	Relational Value task	**Study 1 a) Behavior** • Higher proportion of trials classified as indicative of low relational value associated with increased levels of peer victimization **b) Imaging** • Negative association between levels of peer victimization and functional connectivity between VS-bilat IFC, VS-mPFC, VS- right Put Low> High peer victimization • Positive association between levels of peer victimization and functional connectivity between VS- Left inferior occipital cortex **• Study 2 a) Behavior** • Higher proportion of trials classified as indicative of low relational value associated with increased levels of peer victimization
					**b) Imaging** • Negative association between levels of peer victimization and functional connectivity between VS-bilat IFC in Low> High peer victimization (small volume correction)
Jarcho et al. (34)	Adolescents *N =* 47 (Low Victimized *N =* 20, 10F; High Victimized *N =* 27, 13F)	10–12	Longitudinal wariness and victimization assessment, cross-sectional imaging ROI	fMRI virtual school paradigm	**Receipt of social evaluation:** High victimized: wariness between ages 2 and 7 was associated with activation in right amygdala during unpredicted positive peer evaluation, associated with higher social anxiety symptoms
Kiefer et al. (35)	Adolescents (*N =* 24, 14F)	12–15	Cross-sectional	Cyberball Perfusion MRI study	• Perfusion changes during social exclusion (exclusion vs. inclusion contrast) in the left IFC and sgACC were positively associated with the extent of previous experiences of bullying • Perfusion changes during social exclusion (exclusion vs. inclusion contrast) in the left IFC were positively associated with reported feelings of rejection after task performance
Lee et al. (30)	Adolescents *N =* 23 (all male); High peer verbal abuse *N =* 11, low peer verbal abuse *N =* 12	15–17	Cross-sectional WB+ ROI	fMRI emotional stroop—variation with swear words	**a) Behavior**: No sign differences between groups **b) Imaging**: • Swear words > neutral: high > low verbal abuse L vlPFC, insula • Increased funct connectivity L vlPFC-L hippocampus during swear condition in high>low verbal abuse groups
Lenow et al. (27)	Adolescents (*N =* 32, all F); Victims interpersonal violence *N =* 15, non-victims *N =* 17	12–16	Cross-sectional	Trust game (behavioral only)	• Interaction between Learning Rate and Preference stochasticity (PS): at high PS, learning rate was positively associated with assault frequency
McIver et al. (36)	Adolescents (N45, 36F), from which a) peer victimized (*N =* 15); b) defenders (*N =* 15); c) controls (*N =* 15)	17–19	Cross-sectional ROI	Cyberball	• No significant differences in experienced distress between groups • Exclusion> Inclusion: increased functional connectivity L amyg-ACC and L amyg-R Insula controls > Victimized; defenders had different pattern of functional connectivity with more connectivity in ACC-mPFC during inclusion than exclusion, opposite to what was described for the control and victimized groups • Functional connectivity mPFC-lAmyg in victimized individuals moderates association between victimization and depressive symptoms—these only present when connectivity is positive
Oppenheimer et al. (37)	Adolescents with diagnosis of anxiety disorder (*N =* 36, 19F).	11–16	Cross-sectional ROI	Chatroom Interact Task	• Increased peer victimization mediated the association between right anterior insula activation during social rejection and suicidal ideation (controlling for depressive symptoms)
Perino et al. (38)	Adolescents with conduct problems (*N =* 24, 12F)	13–18	Cross-sectional WB	Cyberball (observer role)	• Bullying scores associated with activation in bilateral amygdala, vStr, Insula, mPFC, PCC during exclusion>Inclusion
Rappaport et al. (28)	Adolescents/Young adults *N =* 56 (26 F), 16 Major Depressive Disorder, 13 MDD NOS	16–20	Longitudinal assessment of victimization symptoms, ERP cross-sectional	Island getaway Doors task ERP study	• Early but not recent peer victimization associated with blunted reward response to social acceptance
Rudolph et al. (22)	Adolescents *N =* 47 (23 non-victimized, 24 victimized, all F)	14–17	Longitudinal peer victimization and symptoms assessment, cross-sectional imaging WB + ROI	Cyberball	• Exclusion> inclusion: Victimized > TD in dACC, amygdala, inferior fusiform gyrus • dACC, sgACC, Insula activation positively associated with higher internalizing symptoms • Association between activation in dACC/sgACC/Insula and internalizing symptoms was partially explaned by a link between activation in these regions and avoidance motivation for victims but not for non-victims
Rudolph et al. (39)	Adolescents *N =* 43 (all F)	14–16	Longitudinal assessment victimization, cross-sectional MRI	Emotion regulation task	• Victimization positively correlated with Amyg-R vlPFC functional connectivity in context negative emotion and negatively correlated with labeling accuracy during negative emotions • Victimization predicted amyg- R vlPFC connectivity in girls with high but not low rejection sensitivity—during ER task • Victimization predicted labeling accuracy in girls with high rejection sensitivity (but not low)
Schriber et al. (23)	Community based sample (*N =* 166, 90F)	16–18	Longitudinal (hostile school environment and familiar support assessment, MRI is cross-sectional and ROI)	Cyberball	• Hostile school environment directly associated with increased social deviance, mediated by activation in sgACC during exclusion contrast in Cyberball task • Activation in sgACC during social exclusion in task was associate with depressive symptoms, deviant behavior and hostile school environment • Family connectedness moderate the mediation model
Swartz et al. (40)	Adolescents from community sample (*N =* 49, 24F)	12–15	Cross-sectional WB + ROI	Emotional face matching task Bullying and victimization are self-report in Qualtrics	• Relational bullying predicted by enhanced activation amygdala during angry faces and reduced during fearful faces, as well as lower activation in rostral ACC to fearful faces • Relational peer victimization associated with lower amygdala to angry faces and fearful faces
Telzer et al. (31)	Adolescents (*N =* 46, all F); Chronically victimized *N =* 25, non-victimized *N =* 21	14–18	Longitudinal victimization and symptomatic assessment, cross-sectional imaging WB	Stoplight Task (twice, pre and post exclusion experiences at Cyberball, only second time inside scanner)	**a) Behavior:** No between-group behavioral differences (risky choices) before exclusion experiences, Vict>nonVict risky choices after exclusion **b) Imaging** • Risky decisions: Vict>nonVict: bilat amyg, vStr, OFC, mPFC, TPJ; Vic < NonVict SMA • Safe decisions: Vict>nonVict mPFC, dlPFC, vlPFC, dmPFC • pass outcomes: Vict>NonVict Striatum; Vict < NonVictbilat Insula **c) Association neural reactivity and antisocial behaviors**: • Risky decisions: bilat amyg, OFC, mPFC, dmPFC, pSTS • Safe decisions: mPFC, dmPFC, TPJ, pSTS, vlPFC, dlPFC
					• Pass outcomes: • reduced act in TPJ, STS, mPFC, dmPFC
Telzer et al. (24)	Adolescents (*N =* 38, all F) Chronically victimized *N =* 21, non-victimized *N =* 17	14–16	Longitudinal victimization and symptomatic assessment, cross-sectional imaging WB+ROI	Social evaluation task	**a) Behavior**: Peer victimization score associated with response time (in-group>out-group) and memory biases Vict>nonVict **b) Imaging:** • positive association between peer victimization scores and increased activation in-group vs. out-group peers in amygdala, vStr, fusiform gyrus, TPJ • This activation was associated with lower social self-esteem and increased internalizing and externalizing symptoms at 9-months follow-up • in-group>out-group activation in vStr, TPJ and amyg is positively associated with in-group memory bias, activation in fusiform negatively with in-group RT bias
de Water et al. (21)	Typically developing (*N =* 52, 17F), subgroup with peer ratings (*N =* 31, 17F)	12–16	Cross-sectional WB	Cyberball Popularity and acceptance rated by classroom peers	**Exclusion > Inclusion: Ball** • vlPFC **• Inclusion:no ball> Inclusion: ball** • vlPFC **• Exclusion> Inclusion: no Ball** • dACC **• Effect own peer status (Exclusion > Inclusion: Ball**) • dACC More> Less accepted **• Effect virtual player popularity** • rACC condition popularity interaction, increased activation by inclusion average popular& exclusion high popular players **• Effect peer x virtual player popularity** • Exclusion by popular> exclusion by average enhanced VStr and mPFC in high vs. average popular participants
Will et al. (89)	Chronically rejected (*N =* 18, 6F) and highly accepted adolescents (*N =* 25, 12F)	12–15	Behavioral longitudinal assessments, imaging cross-sectional WB	Cyberball Dictator game	**a) Behavior**: no between group differences **b) Imaging** (equal treatment excludes>equal treatment includers) • Vict>non-Vict: R lateral PFC, R Caudate • Positive Association with perspective taking—dmPFC, across all participants • Positive Association with regulation problems—L Anterior Insula, pre-SMA/dACC, across all participants
Will et al. (25)	Chronically rejected (*N =* 19, 7F) and highly accepted adolescents (*N =* 27, 13F)	12–15	Behavioral longitudinal assessments, imaging cross-sectional WB	Cyberball	**a) Behavior** • Comparable distress after exclusion in Need Satisfaction questionnaire **b) Imaging** **• Exclusion > Inclusion: ball** • Rejected > Accepted dACC • **Incidental exclusion (Inclusion: no ball> Inclusion: ball)** • Rejected > Accepted preSMA, dACC, anterior PFC

WB, Whole Brain analysis; ROI, Region of Interest analysis; F, Female; Vict, victimized; TD, typically developing; PE, prediction error; R, right; L, left; ICA, Independent Component Analysis; PFC, prefrontal cortex; OFC, orbitofrontal cortex; dlPFC, dorsolateral prefrontal cortex; vlPFC, ventrolateral prefrontal cortex; dmPFC, dorsomedial prefrontal cortex; mPFC, medial prefrontal cortex; SMG, supramarginal gyrus; rACC, rostral ACC; dACC, dorsal anterior cingulate cortex; sgACC, subgenual anterior cingulate cortex; PCC, posterior cingulate cortex; IFC, inferior frontal cortex; SFG, superior frontal gyrus; MFG, medial frontal gyrus; vStr, ventral Striatum; SPL, superior parietal lobe; TPJ, temporo-pareital junction; STS, superior temporal sulcus; RT, reaction time; SMA, supplementary motor area; amyg, amygdala.

## Peer victimization is associated with enhanced sensitivity to social stimuli

Social interactions become crucial during late childhood and adolescence, evidenced by the sharp increase in the relevance and the time they spend with peers (41). Many studies have shown the significant influence of social agents during adolescence on decision-making or risk-taking tasks, both negative (increasing the likelihood of risky decisions) but also positive (they can also reduce the proportion of risky decisions made), which is applicable not only to peers (42–45) but also to relevant adults with whom adolescents maintain significant affective relationships (46, 47). At the neural developmental level, the enhanced sensitivity to social stimuli might be determined by the imbalanced development of the limbic system supporting emotion and incentive processing, relative to that of prefrontal regions supporting regulatory processes. Thus, neural maturation processes of the key regions supporting cognitive control, reward and social processing show a protracted trajectory starting in early childhood and continuing well into adulthood (48–56). The early development of the limbic system relative to the prefrontal cortex facilitates an enhanced individual sensitivity to incentives and emotional contexts (57, 58), thus increasing the risk of severe and long-lasting consequences when an insult occurs during crucial sensitive periods (59–61). Adolescence constitutes indeed the time when the imbalance on the neurodevelopmental trajectories of limbic systems involved in incentive and emotion processing, and prefrontal cognitive control systems is maximal (62–65). Furthermore, hormonal changes including those in crucial stress response systems, Hypothalamus-Pituitary-Adrenal axis (HPA axis) and the Hypothalamus-Pituitary-Gonadal axis (HPG axis), have their peak during this developmental stage (66), thus contributing to the onset or exacerbation of many psychopathological disorders (67). Hence, adolescence is a developmental period where individuals are highly sensitive to social stimuli. This enhanced sensitivity to social stimuli from peers may however play an adaptive role, facilitating the progressive independence of biologically mature individuals from protective parental environments (62), increasing their environmental exploration. It is therefore expected that social stimuli engage brain regions involved in processing of saliency.

The key regions processing salience include both the prefrontal cortex and limbic brain regions. The medial prefrontal cortex (mPFC) plays a key role as part of the neural networks supporting the modulation of amygdala responses to emotional stimuli, thus contributing to emotion regulation processes (68). The key sensitive period for the development of the structure and functional connectivity between amygdala and mPFC lays between late childhood and early adolescence (69, 70). Therefore, disturbances on this developmental phase might result in persistent disruption of emotion regulation skills or emotional reactivity to events. Together with the increased stress-reactivity observed during this phase (71), it would significantly impair their ability to successfully cope with peer victimization situations. Increased stress-induced HPA response in the adolescent brain might affect regions known to be stress-sensitive and that are still under development, in particular amygdala, prefrontal cortex or hippocampus, making the adolescent brain highly sensitive to these stressful situations (72). In support of this suggestion, recent evidence has shown that cortisol response in adolescents mediated the association between cyberbullying and perceived stress (73), as well as the association between early victimization experiences, subsequent abnormal cortisol response and reduced area in prefrontal cortex (74).

Indeed, because of this already heightened sensitivity, experiences of peer victimization and bullying may lead to pervasive, deleterious consequences. Theories like the Sociometer Theory (75) or the Need to Belong (76) postulate the existence of internal monitoring systems that interpret environmental signals of acceptance or rejection during social interactions with peers. These signals provide the individual a sense of belonging and the relational value with respect to the group. This need to belong is already present in very young children (77), with emotional, cognitive and behavioral detrimental effects (such as emotional distress; symptoms of depression, anxiety or irritability; hypervigilance for social cues or persistence/tolerance of abusive behaviors) when not fulfilled (76). Individuals would therefore be innately inclined to establish a number of interpersonal relationships that would need to have a positive, stable and significant character. Consequently, social deprivation becomes a punishment and positive social contact a reinforcer (76). Experiences of peer victimization and bullying during adolescence can trigger the need to belong to a group, which is not satisfied and enhances the social monitoring system (including regions typically processing social salience, mentalization or affective processing) (24). This might suggest increased sensitivity and hypermonitoring of social signals, with the goal to identify potential avenues to recover the homeostatic state where those needs are met, increasing behavior that has the potential outcome of being accepted by the group.

Preliminary evidence supporting this suggestion comes from a recent study using a minimal group approach (24). In this case, participants were included in a group and shown pictures that could be (1) pictures of other members of the same group (in-group), (2) pictures from participants who are in a separate group (out-group) or (3) pictures of participants who have not been assigned to any group (neutral). After establishing the minimal group, female adolescents (aged 14–16) who suffered severe long-term victimization performed a social evaluation task inside the scanner, where they were asked to indicate to each of the facial stimuli simply whether they liked them or not. Afterwards, they were presented with images of new faces together with faces used in the establishment of the minimal group, and they had to indicate whether they had already seen that face or not. Victimized girls showed enhanced activation in regions supporting social monitoring processes including the amygdala, ventral striatum (vStr), fusiform gyrus and temporo-parietal junction (TPJ) during assessments of in-group vs. out-group pictures. The higher social sensitivity is suggested by the association between imaging and behavioral results, as activation in amygdala and vStr was associated with implicit behavior bias toward the in-group, with increased reaction time (RT) and memory to in-group pictures. These results would support hypothesis of enhanced sensitivity to social stimuli after experiences of peer victimization, results that are further strengthened by reported increases of cortical thickness in the fusiform gyrus of victims of bullying relative to non-victims (78). Thus, the structural and functional abnormalities in this key area might indicate enhanced sensitivity to facial expressions.

Other studies have utilized paradigms assessing facial emotion processing to investigate a potential enhanced sensitization to social stimuli after experiences of victimization. Such a task was used to investigate brain function in female adolescents victims of interpersonal abuse (aged 11–17), where participants were presented with neutral or fearful faces and they had to press for the gender of the face (20). The study found increased activation in the dorsal anterior cingulate gyrus (dACC) and the anterior insula during processing of fearful expressions in those participants who had a high exposure to victimization relative to those with low or no exposure to victimization experiences (20).

One of the most commonly used tasks to assess neural and behavioral responses to social exclusion is the Cyberball task (79). In that task, participants are typically induced to play an interactive ball-tossing game with 2 other players (real or pre-programmed). They usually play two rounds, one where they are included in the game and receive the ball about 1/3 of the trials, and a second round where they are ostensibly left out of the game by the other two players. By contrasting the inclusion vs. exclusion blocks the paradigm aims to investigate social exclusion.

Several recent studies have investigated the neural correlates of social rejection in victimized adolescents. Thus, victimized adolescents (14–17 years of age) whose status had been assessed longitudinally over the previous 7 years showed enhanced activation compared to non-victimized peers in the dACC, amygdala, and fusiform gyrus in the exclusion > inclusion comparison (22). In another study in a slightly younger sample (aged 12–15) participants had been assessed once a year on their social status in the classroom between the ages of 6 and 12 (25). The version of the task used allows for the comparison not only of exclusion vs. inclusion rounds, but also investigated the incidental exclusion events (inclusion: no ball vs. inclusion: receive ball). Despite their comparable levels of stress reported after social exclusion, participants who had experienced chronic rejection showed enhanced activation relative to those without such experiences in dACC during exclusion relative to inclusion rounds, as well as increased activation in dACC and anterior prefrontal cortex (PFC) during incidental exclusions (25).

Another cross-sectional study used the Cyberball task to investigate behavioral and neural responses to social exclusion as a function of the popularity and acceptance status of both participant and interacting partner (21). The authors differentiate between individuals who are accepted (i.e., whether they were liked or not in the classroom, rated by classroom peers) and popular (i.e., popularity rates, not necessarily the most liked in the classroom group). Thus, typically developing adolescents (12–16 years old) played a version of Cyberball in which both themselves and the opponents could be high or average accepted and high or average popular. Most prominently, exclusion enhanced activation relative to inclusion conditions in the dACC and the ventrolateral prefrontal cortex (vlPFC). Participants who were themselves more accepted showed enhanced activation in the dACC during exclusion (vs inclusion:no ball) condition. Participants' own popularity was positively associated with increased activation in vStr and medial prefrontal cortex (mPFC) when they were excluded by highly popular but not by average-popular virtual players (21). This contrasts with the results from the Will et al. study (25), where the enhanced activation in dACC during exclusion trials was observed in chronically rejected adolescents rather than in those without relevant rejection experiences. However, these studies differ in two key elements. One is that while Will et al. include chronically rejected adolescents, participants in the de Water et al. study included a community sample of students who were high or average accepted/popular. In addition, this study required the knowledge about the social status of the opponent to be integrated in relation to one's own. In the case of highly accepted participants, this might have led to conflict detection, to be potentially resolved by the increased engagement of the dACC. Finally, it must be noted the difference in the contrast used, as Will et al. used the Exclusion>Inclusion Ball contrast whereas De Water et al. used the Exclusion > Inclusion No Ball contrast. This is an important differentiation that they include given that participants who are highly popular may show some antisocial behaviors.

The impact of experiences of bullying and peer victimization on neural responses to social rejection has also been investigated combining perfusion brain imaging methods and the Cyberball paradigm (35). Previous experiences of bullying were associated with increased perfusion in key regions for social pain processing including the subgenual anterior cingulate cortex (sgACC) and left inferior frontal cortex (IFC). Furthermore, the authors observed a positive association between perfusion in the left IFC and self-reported feelings of rejection. Thus, the evidence from this study provides further support to the hypothesis that experiences of bullying and peer victimization enhance sensitivity of social pain/social processing systems, potentially related to increased mentalizing and rumination processes that increase individual sensitivity to signals of social exclusion (35).

Not only altered regional activation has been described, but also abnormal functional connectivity. Thus, recent studies have shown that adolescent girls (14–16 years of age) exposed to high peer victimization had to either passively observe emotional faces, or choose one of the two words to label the emotion shown by the facial stimulus. The victimized group had stronger positive connectivity between right vlPFC and amygdala (indicative of worse emotion regulation abilities) in those cases with high sensitivity to social rejection (39). Similarly, altered functional connectivity was recently reported in a study using the Cyberball (across all exclusion and inclusion conditions) (36). Reduced connectivity between left amygdala and right insula as well as between left amygdala and ACC was observed in peer victimized adolescents relative to non-victimized peers (36). This raises the possibility that peer victimization may have disrupted the maturational process by which the mPFC downregulates amygdala activation when facing emotional stimuli (80–82).

The evidence also suggests that altered brain function is not only present in victims but also in those perpetrating bullying behaviors. In a recent study, a community sample of boys (aged 12–15) performed a face matching task inside the scanner and provided additional self-report measures on bullying and victimization behaviors (40). While high self-reported victimization was associated with high amygdala activation to both angry and fearful faces, bullying behaviors were associated with heightened amygdala response to angry faces and reduced response to fearful faces. In addition, increased activation in the genual ACC to fearful faces was associated with less bullying behaviors.

Similarly, Perino et al. (38) conducted a study in which adolescents with conduct problems (aged 13–18) watched a passive version of the Cyberball where others were excluded (bullied) or included during the game. Self-rated bullying behaviors were positively correlated with differences in activation in the mPFC, insula, vStr and amygdala when watching exclusion relative to inclusion rounds. While the authors interpreted these results as indicating that bullying is associated with neural activation during situations where social hierarchy cues are salient, these results could also be indicative of enhanced salience of emotionally relevant stimuli. In line with this, another study showed that enhanced activation during social exclusion blocks in the Cyberball task is associated with the presence of subsequent problematic behaviors (23). Thus, increased activation in the sgACC during exclusion compared to inclusion blocks was shown to mediate the association between past experiences of hostile school environment (experienced between 1 and 3 years before the scanning session) and subsequent social deviant behavior, measured 6 months after scanning session and defined as the presence of externalizing behaviors and affiliation with deviant peers (23). However, it is important to note that the presence of relevant family support modulated this effect, mitigating the impact of the hostile school environment (23).

In summary, the most recent evidence suggests that individuals who had been exposed to peer victimization or bullying might show hypervigilance or enhanced sensitivity to social stimuli and social valuation. This could be related to their need to belong to social groups, and enhance activation in salience networks including the sgACC, dACC, anterior insula, dorsomedial prefrontal cortex or amygdala, triggering potentially distressing emotional responses and increasing their risk to psychopathology.

## Altered reinforcement learning and reward processing

An additional potential mechanism linked to the behavioral consequences of peer victimization is altered reinforcement learning and reward responses. The study from Cisler et al. (20) using reinforcement learning models provides interesting evidence supporting this hypothesis. They used an interpersonal trust game and a non-social three-armed bandit control task to investigate brain function in female adolescents victims of interpersonal abuse and how brain activity patterns might be associated with the persistence of PTSD symptoms (20). Participants (aged 11–17) had to choose one out of three people in whom to invest 10$, and they would receive either 20$ or 0$ in return from the investee. The non-social version of the game used pictures of three houses instead of human faces, and winning 20$ vs. 0$ was dependent on whether the door would open or not. This study found that compared to non-victimized adolescents, increased exposure to victimization was associated with increased activation in the dACC, insula as well as with reduced activation during those trials where the expected reward was not delivered (negative prediction errors) (20). This effect was observed both for social and non-social incentives, albeit with slightly stronger effect in the social context (20). Thus, victimized adolescents showed increased activation in key regions processing salience including the dACC, insula and amygdala during facial emotion processing, whereas during negative prediction errors these areas showed underactivation compared to healthy control individuals (20).

Similarly, using event related potentials (ERP), it has been shown that peer victimization in late adolescents (aged 16–20) was associated to blunted reward responses to monetary reward, but even more so to social rewards (28). Moreover, a recent EEG study in healthy young adults (18–25 years of age) showed the association between blunted reward response during feedback in a forced-choice task that was associated with self-reported relational victimization but not with physical victimization (26). Although not including brain imaging, it is worth mentioning the study from Lenow et al. (27). The authors used computational modeling analysis and a modified version of a trust game. In their study, female adolescents (12–16 years old) victim of early life interpersonal violence had to decide which of the 3 potential faces was most trustworthy (27). Not only did victimized girls had a lower learning rate than those in the control group, but learning rate was shown to interact with preference stochasticity, by which girls with higher learning rate also had higher stochasticity rates. Thus, victimized individuals might update the assigned reward value based in the most recent history, ignoring previous potentially contradictory evidence which might lead to situations where they are highly vulnerable. In addition, these results indicate random changes in their trustworthiness preferences which, while adaptive in highly volatile environments, might be suboptimal in more realistic, stable environments (27). These results are in line with recent evidence on adolescents with a history of maltreatment during an associative learning task (83), with initial beliefs of reward being more volatile and random, and reduced ability to learn about the reward pattern and to use the information about rewards adequately (83). While such reward beliefs could be adaptive in households with high volatility, they might in turn lead to behavioral difficulties. This study furthermore showed that problems in associative learning might partially account for the link between early adversity and behavioral problems.

Thus, victimized adolescents might show abnormal reward and punishment processing which might interfere with their ability to make decisions, both in the presence of monetary incentives but also on social contexts. Individuals subject to victimization and bullying experiences might therefore show reduced ability to learn from feedback, being less able to anticipate the consequences of their actions (for example, rewards or losses in a lab-based paradigm). In addition, they might experience stronger emotional reaction to losses/rewards, even at the time of the cue, leading to suboptimal decisions. Taken together, these results have interesting implications that might also provide some preliminary insight on the mechanisms by which victimization occurs and perpetuates. Their difficulties to learn from the previous reward history (27), and their blunted neural response in anticipation or response to reward (26, 28) or to the absence of reward when this is expected (20) might lead to behavioral adaptations increasing the individual vulnerability to internalizing symptoms like depression or anxiety, or perpetuating abusive or toxic relationships.

## Peer victimization is associated with an increased need to recruit regulatory systems

The influence of emotions on social decision making processes is well established (84, 85). The enhanced sensitivity to social stimuli observed in adolescents who have experienced peer victimization might interfere with the implementation of self-regulation, controlled processes required when facing relevant social situations.

Studies investigating differences in impulsive and risky behavior have provided some support to this respect. Telzer et al. (31) investigated the association between previous experiences of victimization in female adolescents and subsequent impulsive, risky behavior. Participants (aged 14–18) performed a simulation driving task, where at the road crossings they saw a yellow traffic light and could make a risky decision and try to cross before the light went red or make a safe decision and stop. They played the task before and after a classic Cyberball game, the second time inside the scanner. While the two groups did not differ in their initial task performance, the group of chronically victimized girls showed higher proportion of risky decisions after the exclusion experience in the Cyberball. Furthermore, they had higher activation of cognitive control regions during “safe” choices (vlPFC and dorsolateral prefrontal cortex - dlPFC-) interpreted as a result of the need to make stronger effort to implement control over their behavior. During risky decisions on the other hand there was increased recruitment of affective sensitivity (amygdala, vStr and orbitofrontal cortex) and social cognition (superior temporal sulcus -STS- and TPJ) regions, which in addition were associated with aggressive behaviors in everyday life. The authors suggest that would be interpreted as taking risk behaviors to satisfy the need to belong, as a way to get peers acceptance. A recent study investigated the association between exposure to violence in adolescence (lifetime and in the last year) and brain activation and performance during a modified version of a basic motor response inhibition task, the Go/NoGo task (29). Adolescents (9–14 years old) showed that increased exposure to violence (both types) was associated with reduced activation in key regions of inhibitory function network, including the dACC or the mPFC, which in the case of violence during last year also included superior parietal regions. Furthermore, the reduced activation in the dACC and posterior parietal areas was associated with progressive performance deterioration, with latency increasing with time on task. Hence, the authors suggest that exposure to violence affects basic self-regulation function. Similarly, a study in adolescents (15–17 years old) exposed to peer and parental verbal abuse showed that during the performance of an emotional Stroop task that included swearing words, there was enhanced activation during their swearing>neutral condition in the left vlPFC and enhanced functional connectivity between left vlPFC-hippocampus, which might be interpreted as a need to implement higher cognitive control due to the enhanced sensitivity to the aversive stimuli (30).

Some recent evidence suggests that the enhanced sensitivity to social stimuli may trigger the additional recruitment of key regulatory regions, such as the dlPFC to implement self-regulation processes. Different studies have shown that activation in this region varies in children and adolescents during social exclusion situations as a function of their previous experiences with school peers. Thus, a longitudinal study assessed whether primary school children (aged 8–12) were high- or low-preferred over 3 years prior to performing the Cyberball task in the scanner. Despite the lack of between-group differences in their reported distress after exclusion experiences, boys who were low relative to highly preferred showed increased activation in bilateral dlPFC and right supramarginal gyrus during exclusion (relative to inclusion:others) contrast (32). The authors suggest their finding of enhanced activation in the supramarginal gyrus could be associated with the reported involvement in this region in internal blame attribution (86). This would link with the idea that adolescents lack the adult cognitive biases to protect their self-esteem after experiences of social rejection, thus blaming themselves after such exclusion processes. Such interpretation is also consistent with the suggestion of enhanced recruitment of dlPFC regions being required to implement emotion or behavioral regulation processes. This is also in line with evidence from typically developing populations showing the role of the dlPFC to regulate emotions and aggressive responses after socio-emotional feedback (87, 88). Indeed, longitudinal increases in activation of the dlPFC have been associated with a reduction in aggressive behaviors especially after receiving negative social feedback (87, 88). Therefore, recent available evidence supports the role of the dlPFC as key to self-regulate responses after social exclusion experiences both in typically developing (87, 88) and vulnerable children (32).

A recent study used the Social Evaluation Paradigm (33), where adolescents were presented with pictures of same-aged peers and had to indicate (a) how they liked each of them and (b) how they anticipated each of those peers would rate (i.e., like) the participant back. Neural responses to the subset of pictures rated positively (i.e., liked) by the participants in (a) and where a positive evaluation was anticipated (i.e., the participant anticipated that peer would also like him/her back, high relational value), were compared to those that were positively rated in (a) but negatively in part (b) that is, the participant liked the peer but anticipated that peer would not like him back (low relational value) (33). There was a significant positive association between the number of trials with low perceived relational value and levels of self-reported experiences of peer victimization, as well as an association between peer victimization experiences and reduced functional connectivity in the contrast of low>high relational value between the VStr-bilateral IFC, mPFC and right putamen, together with increased functional connectivity between VStr-left inferior occipital cortex (33). Comparable effects were observed in a smaller sample of females with higher levels of peer victimization. As suggested by the authors, the altered connectivity patterns might be signaling an increased need to implement self-regulation processes, given the role of the IFC to downregulate striatal responses to appetitive/salient stimuli (33).

Another study provides further evidence on the additional recruitment of self-regulation regions required in chronically victimized adolescents (89). Participants (12–15 years old) experienced first the exclusion and inclusion phases of the Cyberball, to next perform a Dictator game, where they have to decide how to split some monetary units with those individuals who had previously accepted or rejected them during the Cyberball. This provided participants with the opportunity to retaliate and punish those who previously rejected them. Behaviorally, victimized and non-victimized participants did not differ on their unequal choice distribution for excluders. They also did not differ on brain regions engaged during punishment of excluders. However, during trials where they chose not to punish those who previously excluded them (compared to choices not to punish those who included them), chronically rejected individuals show enhanced recruitment of the lateral PFC and caudate, which highlights the need to recruit additional control regions to successfully implement self-regulation. In addition, positive associations between activation in this contrast in the anterior insula and dACC and parent-reported behavioral regulation problems were observed across all participants.

To sum up, individuals exposed to victimization experiences may need to engage behavioral and emotional self-regulation networks to a larger extent than non-victimized individuals in order to reduce the emotional distress or the aggressive reactions triggered by the increased sensitivity to social stimuli. However, whether this can be considered as part of a potential resilience mechanism would need further research. It could be that those individuals who are not able to additionally engage self-regulation regions cannot refrain aggressive behaviors, thus becoming bully-victims. Given the scarce evidence on the topic and the instability in the trajectories of individuals in a bully-victim role (90), only longitudinal brain imaging studies can clarify this aspect.

## Association with psychopathology: Vulnerability and modulating factors

The increased risk for internalizing and externalizing disorders subsequent to experiences of peer victimization has long been highlighted (3, 91–93). While both relational and physical victimization have been associated with increased externalizing problems, individuals subject to physical victimization typically show increased aggressive behaviors, whereas victims of relational victimization more often develop internalizing problems (3). Despite the idea that physical victimization might lead to more aggression [according to the “cycle of violence” theory (94)] and that relational or emotional victimization is more strongly associated with internalizing symptoms, a recent study shows this is not the case, with every type of victimization being similarly associated with general psychopathology and invariant of gender (95).

Recent studies have provided evidence on how changes in brain morphology may mediate the association between peer victimization and psychopathology. Thus, experiences of peer victimization have been associated with reduced volumes in the medial orbitofrontal cortex both in adolescents at high risk of psychosis and healthy participants (96), with structural abnormalities in the striatum which mediated the presence of generalized anxiety symptoms (97) and with volumetric changes in the nucleus accumbens mediating the increase in symptoms of depression during adolescence (98). Similarly, adults with symptoms of depression and history of bullying between 13 and 17 years of age showed altered white matter integrity, with increased fractional anisotropy measures in the superior corona radiata, which are hypothesized to be subsequent to hyperactivation in the fear network (99).

Some of the studies reviewed provide further evidence to help improve our knowledge of the mechanisms underlying such mediating role and report associations between altered function in key brain regions and psychopathological symptoms, internalizing in most of the cases. Thus, enhanced activation in salience and social processing regions in victimized adolescents (vs. non-victimized peers) during exclusion (vs. inclusion) conditions in the Cyberball task (dACC, sgACC and anterior insula) was significantly associated with increased internalizing symptoms (across all participants) (22). During a social valuation task, victimized girls showed enhanced activation in social monitoring networks including the amygdala, vStr, fusiform gyrus and TPJ during assessments of in-group vs. out-group pictures, activation that was inversely associated to self-esteem across schools years, and positively associated with internalizing and externalizing symptoms 9 months later (24). Thus, higher social sensitivity and need to belong increased vulnerability to subsequent psychopathology. In addition to alterations in local activity, positive functional connectivity between mPFC and amygdala was significantly associated with depressive symptoms (36). However, the authors report a reduced functional connectivity across both inclusion and exclusion blocks of the Cyberball task was observed between the left amygdala and the ACC. This raises the possibility that peer victimization may have disrupted the maturational process by which the mPFC downregulates amygdala activation when facing emotional stimuli (80–82). On the other hand, in the study from Lee et al. (30) the increased recruitment of vlPFC and enhanced vlPFC-hippocampus connectivity was associated with less severe anxiety and depression symptomatology, which they interpreted as a reduced impact at the psychopathological level in those able to implement stronger self-regulatory processes. However, there is also evidence of a lack of association between differences in brain activation and psychopathology, as it is the case of the study from Cisler et al. (20). In addition, the experience of peer victimization has been shown to mediate the association between enhanced activation in the anterior amygdala and suicidal ideation in adolescents with depression symptoms (37). Thus, peer victimization might not only be linked to negative mental health outcomes but also worse their severity or adverse consequences for individuals with mental health symptomatology.

It is important to note here the key mediator role that the coping strategies implemented by victimized adolescents might play. Thus, the association between activation in the dACC and sgACC and internalizing symptoms were partially mediated by avoidance strategies in victimized youth, whereas the association between insula activation and symptoms was significant overall and did not differ between the two groups (22). These findings are indeed in line with recent evidence on children who experienced early adverse threatening events, whose increased internalizing symptoms observed at adolescence were mediated by the use of avoidance strategies (100). Hence, these adverse, threatening social experiences might have sensitized neural systems processing social signals and increased the risk for internalizing psychopathology in those individuals who use maladaptive coping strategies.

While studies have mostly focused on the association between experiences of peer victimization and subsequent depression or anxiety symptoms, it is important to note that pre-existent psychopathology or symptoms might lead to increased risk for further victimization (91, 93). Similarly, the most recent studies show how other aspects such as increased sensitivity to social rejection or wariness that have also been typically associated as consequences of peer victimization experiences can indeed constitute significant vulnerability factors. A recent study used the virtual school paradigm to investigate wariness as a potential vulnerability factor by which exposure to peer victimization might contribute to abnormal neural function (34). In this paradigm, participants receive positive, negative or neutral feedback from a virtual peer with a reputation of being “mean”, “nice” or “unpredictable”. The results show that in highly victimized children, wariness rated by the parents between ages 2 and 7 was associated with higher amygdala activation during unpredictable positive peer evaluation when they were 11 years old, which was associated with concurrent social anxiety (34). Thus, wariness in early childhood might constitute a vulnerability factor to subsequent social anxiety when faced with social stress situations. Similarly, adolescents girls exposed to high peer victimization showed stronger positive connectivity between right vlPFC and amygdala (indicative of worse emotion regulation abilities) only in those cases with high sensitivity to social rejection (contrast: facial emotion labeling vs. passive watching) (39). While the mediating role of high rejection sensitivity was detrimental in those cases who were exposed to high peer victimization, exposure high rejection sensitivity was associated to better emotion regulation (increased negative functional connectivity between amygdala and rVLPFC) in cases with low peer victimization. These results would suggest that individual differences might be protective or risk factors as a function of the environmental experiences of the individual.

Not only the presence of previous psychopathology has been shown as an additional factor of vulnerability to victimization (34, 91, 101). A similar role has been proposed for cognitive function. Thus, it has been recently suggested that the presence of cognitive deficits could be conceptualized as potential pre-existent risk factors, which could in addition complicate intervention response (102). Also deficits in response inhibition have been hypothesized as a potential vulnerability factor by which children who suffer peer victimization might display later bullying behaviors (103), and executive function measures have been suggested to moderate the association between early victimization and subsequent aggressive behaviors (104). Therefore, community studies would be helpful in order to clarify when and how this enhanced sensitivity to social stimuli and social rejection appear, as well as the altered reinforcement learning. We would be able to detect vulnerable individuals early in time, which might help to provide them with the potential support or tools to better navigate their social environments at a sensitive developmental phase. School based intervention programs on social skills could be feasible cost-effective possibilities to address this.

On the other hand, the previous experience with supportive others (family, friends) might serve a protective function, mitigating the deleterious impact of peer victimization experiences (23, 105–107). Recent evidence has also shown that the association between cyberbullying and well-being in adolescence might be influenced by the level of social connectedness (108). One potential hypothesis is that previous experiences with supportive social networks might provide some sense of belonging, fulfilling this need to some extent. It might also serve as scaffolding for the development of potential defensive cognitive biases, similar to those observed in adults. Thus, the discrepancy between the negative information received by the peer rejection and the image of the self is resolved in adults by cognitive biases such as externalizing the negative feedback received or updating their opinions of the peers after their rejection (109–111). These strategies allow them to protect their self-view after experiences of peer rejection, helping reduce or minimize their negative impact, severity or duration. Such strategies are not yet in place in children and adolescents, as they show a tendency to internalize the ground for peer rejection when that happens and to maintain their views of peers after these have rejected them (110). Some preliminary evidence suggests the right supramarginal gyrus as enhanced in adolescents who experience social rejection (32), region that has been suggested to be involved in internal blame attribution. Intriguingly, recent evidence using social networks analyses in combination with structural brain imaging has shown a higher degree of similarity in brain morphology of adolescents who are close friends than in unrelated distant friends (112). Thus, studies investigating the potential role of peer support in the mitigation of the adverse impact of experiences of peer victimization at the neural level would be needed.

There are some potentially relevant factors that are however typically not reported and should be considered in light of recent available evidence, such as race or cultural background of the community, which may influence the neurodevelopment of the social monitoring system and in turn contribute to the increased vulnerability to negative peer interactions. An interesting study has shown that persistent experiences of social discrimination also have long term consequences that affect social behaviors differently depending on race (113). White and Black South Africans who had experienced the Apartheid were exposed to clips depicting victims (forgiving/unforgiving) and perpetrators (apologetic/unapologetic) of apartheid crimes. While previous experiences of social adversity were associated with reduced compassion across participants, social discrimination had differential effects on neural activation, potentially due to the fact of different types of social discrimination experienced. Thus, Black participants experienced social discrimination due to race reasons and this was associated with increased activation in social saliency and pain processing networks, whereas White participants who experienced social discrimination mostly due to income level, weight or gender reasons showed undifferentiated amygdala activation. This suggests that not only race but also the structural and cultural differences of the societies leave their imprint at the neural level, at least partially shaping the processing of social stimuli and therefore determining socio-emotional development. While these structural differences at societal level are difficult to tackle, increased awareness of their impact should help improve our understanding on how the social context of the individual determines his socio-emotional development, potentially increasing their vulnerability to peer victimization or bullying experiences.

## Conclusions

The most recent evidence on the neurobiological correlates of the social and emotional impact of peer victimization and bullying experiences suggests that these experiences increase an already sharpened individual sensitivity to social exclusion or rejection, enhancing neural responses to social stimuli and social valuation processes. This enhanced neural response to social stimuli might require the additional recruitment of self-regulation networks in order to successfully implement controlled responses to emotions and behaviors, which might in turn contribute to the development of deviant behavioral adaptations and psychopathology. In addition, altered reward and reinforcement learning processes may contribute to the unsuccessful behavioral adaptation and perpetuate the display of inadequate behaviors.

The studies reviewed here provide some insights on the potential mechanisms by which peer victimization negatively impact socio-emotional development. While the presence of some factors like familiar or peer support might mitigate these negative effects, the presence of risk factors such as pre-existent psychopathology or enhanced sensitivity to rejection may increase their vulnerability to further abuse or victimization. However, the role of other factors such as age, gender, frequency and intensity of the peer victimization event(s), previous positive and negative social experiences or the presence of potential school support which are likely to moderate the consequences of victimization has yet to be investigated.

One key aspect to consider is the cross-sectional character of the brain imaging data here reported. While a significant strength of some of the studies reviewed includes some longitudinal report on victimization levels, with varied sources of information and not restricted to self-informant (22–25, 28, 31, 32, 34, 39, 89), the imaging data has typically a cross-sectional nature. It is therefore not possible neither to unequivocally disentangle the factors driving the association between experiences of peer victimization and the reported altered brain structure, function or connectivity, nor to exclude that just as it happens with psychopathology, the pre-existence of abnormal brain structure or function might constitute a potential risk factor that increases the likelihood of becoming subject to peer victimization. Studies where negative peer experiences are assessed retrospectively have the associated risk of potential recall bias in terms of the timing, frequency and severity of the recalled event, and therefore may not accurately identify the potential impact at different stages of brain development, where sensitive periods might confer differential risks. However, it is only by collecting brain imaging data in parallel that we may identify the relative contribution of these experiences and the identification of factors that may contribute to individual's vulnerability and resilience. Only by conducting longitudinal, population-based studies can we improve our knowledge on these areas.

Particularly interesting are the findings on impaired reinforcement learning and reward processing. Conducting further research in this area has the potential to improve our understanding on the mechanisms by which these negative early experiences can increase the risk for psychopathology, especially internalizing symptoms, and to perpetuate behaviors that expose the individuals to further victimization. Thus, future studies should better delineate the extend and variability of these difficulties and the potential benefits of different interventions. These could focus on generating alternative behavioral patterns and identifying potential cognitive distortions, using behavioral management or problem-solving techniques.

The specific characteristics of the experience may also differentially impact on brain systems. Models investigating the impact of early experiences on development have taken different approaches. While some have considered that adverse experiences might have a cumulative effect (114), such consideration would assume a comparable impact of the different events due to similar dysregulation on the stress-response system. Other models have suggested that the type of event experienced would influence the individual's stress response leading to different behavioral and clinical presentations (115). Dimensional models propose the differential impact of early adverse events as a function of the type of experience, differentiating between neglect/deprivation and abuse/threat (10, 116, 117). While it might wise to assume that these models could also be applicable to experiences of peer victimization, the reviewed evidence is not unequivocal in this respect and further research is required to test whether and how such models would apply depending on the type of experience.

Two areas of research are promising fields that might help to improve our understanding on the neural mechanisms of peer victimization in adolescence. The first one is the use of connectomics. This method uses graph theory to help quantify, visualize and improve our understanding of brain network organization, especially in terms of the whole-brain integration of structural and functional connectivity at the system level (118). It conceives of the brain as a network [the ‘connectome', (119)], composed by a set of nodes (brain regions) linked by edges (axonal projections) (120, 121). The term “developmental miswiring” has been coined to refer to the disruption of normative development, which might increase individual vulnerability to neuropsychiatric disorders (122). While there is some preliminary evidence on how network reconfiguration might modulate resilience or susceptibility to psychopathology (123), studies specifically addressing the impact of peer victimization on neural networks might significant contribute to improve our understanding on vulnerability and resilience processes. In addition, the use of graph theory methods constitutes a promising avenue to implement social network analyses (112). Such studies have the potential to improve our understanding on the mechanisms by which social, family or peer support might contribute to protect or mitigate the adverse effects of peer victimization experiences. However, the available evidece to date has a cross-sectional nature and therefore further evidence including longitudinal studies is currently required.

It is finally important to mention that assessing the impact of victimization, bullying or social exclusion/rejection processes in adolescence in the scanner necessarily implies a virtual interaction. Although this might on one hand reduce the generalization of the findings into real life situations, we have to consider how much media use, and especially in situations like the current COVID pandemic might influence the way adolescents relate and present to others using social media (124). An increased online contact, which is difficult to control together with the developmental need of high social contact with peers increases the risk of been cyberbullied. Thus, it is important to highlight the need to further investigate cyberbullying and the mechanisms underlying it adverse consequences (125). It therefore constitutes a relevant aspect to be further studied, given that social media use involves key processes that are still under development during childhood and adolescence including reward and emotion-based processing, emotion regulation or mentalizing. Improved knowledge of the medium and long-term impact on these processes might be crucial to investigate well-being and to identify vulnerable individuals and provide measures to protect them from the potential negative consequences (124).

This selective review has focused on the neural and cognitive mechanisms by which social interactions and socio-emotional development may be affected after peer victimization and bullying experiences. However, it cannot be overlooked that other consequences are also commonly observed such as altered inflammatory and immune responses or somatic symptoms, to mention some. In conclusion, the most recent evidence on experiences of victimization and bullying during adolescence suggest that, just as the impact of early adverse events, they have severe and long-lasting consequences on socio-emotional development, interfering with typical neural development. The consequences might however differ depending on a number of factors including the age and gender of the participant at the time, or the type, intensity, duration or circumstances of the adverse event. Finally, individual differences in vulnerability and resilience should be considered. An improved understanding of the neurobiological consequences of exposure to such situations might help identify individualized intervention targets.

## Author contributions

AC: conceptualization, literature search, and manuscript writing.

## Funding

The author was supported by funding from the Jacobs Center for Productive Youth Development to the Department of Economics, University of Zurich, for a separate project.

## Conflict of interest

The author declares that the research was conducted in the absence of any commercial or financial relationships that could be construed as a potential conflict of interest.

## Publisher's note

All claims expressed in this article are solely those of the authors and do not necessarily represent those of their affiliated organizations, or those of the publisher, the editors and the reviewers. Any product that may be evaluated in this article, or claim that may be made by its manufacturer, is not guaranteed or endorsed by the publisher.
